# Punicalagin Inhibits the Growth and Proliferation of Ovarian Epithelial Adenocarcinoma Cells Via Apoptosis and Autophagic Cell Death

**DOI:** 10.1002/jbt.70908

**Published:** 2026-05-14

**Authors:** Zeeshan Ahmad Bhutta, Cho‐Won Kim, Hwayoung Na, Hong Kyu Lee, Kyung‐Chul Choi

**Affiliations:** ^1^ Laboratory of Biochemistry and Immunology, College of Veterinary Medicine Chungbuk National University Cheongju Chungbuk Republic of Korea; ^2^ State Key Laboratory of Vaccines for Infectious Diseases, Xiang An Biomedicine Laboratory, Center for Molecular Imaging and Translational Medicine, School of Public Health Xiamen University Xiamen China; ^3^ Division of Endocrinology, Children's Hospital Boston Harvard Medical School Boston Massachusetts USA; ^4^ Department of Companion Animal Health, College of Biomedical Science & Health Inje University Gimhae Republic of Korea

**Keywords:** apoptosis, autophagy, carcinoma, ovarian cancer, punicalagin

## Abstract

Ovarian cancer remains a leading cause of gynecologic cancer‐related mortality and is frequently associated with therapeutic resistance. Punicalagin (PCG), a pomegranate‐derived polyphenol, has demonstrated anticancer activity in multiple tumor models, however, its effects in ovarian cancer require further clarification. This study evaluated the anti‐proliferative and anti‐migratory effects of PCG in two biologically distinct ovarian cancer cell lines, OVCAR‐3 and SKOV‐3. PCG treatment (6.25–200 µM) significantly reduced cell viability and colony formation in a dose‐ and time‐dependent manner. Migration‐associated behaviors were suppressed in wound‐healing and transwell assays, accompanied by modulation of epithelial–mesenchymal transition markers, including increased E‐cadherin and decreased N‐cadherin expression. PCG increased reactive oxygen species (ROS) levels and reduced mitochondrial membrane potential in both cell lines. Annexin V/propidium iodide analysis demonstrated increased apoptotic cell populations, with elevated BAX expression. Autophagy‐related changes, including LC3‐I to LC3‐II conversion and acridine orange–positive vesicles, were observed in OVCAR‐3 cells but not in SKOV‐3 cells. Collectively, these findings indicate that PCG exerts anti‐proliferative and pro‐apoptotic effects in ovarian cancer cells and is associated with oxidative stress and mitochondrial dysfunction. Further studies are required to define the mechanistic contribution of ROS and autophagy and to evaluate translational relevance in vivo.

## Graphical abstract

Punicalagin targets ovarian cancer via multiple anti‐cancer mechanisms. This graphical abstract summarizes the key findings of the study on the effects of Punicalagin (PCG), a pomegranate‐derived polyphenol, on ovarian cancer cells (OVCAR‐3 and SKOV‐3). The “Input” panel shows PCG, which is then demonstrated to elicit its anti‐cancer effects through four interconnected cellular mechanisms: (1) PCG inhibits cell proliferation and colony formation while suppressing migration and modulating epithelial‐mesenchymal transition (EMT) markers (increasing E‐cadherin, decreasing N‐cadherin). (2) Treatment with PCG induces oxidative stress, indicated by increased reactive oxygen species (ROS) levels. (3) PCG causes mitochondrial dysfunction, characterized by reduced mitochondrial membrane potential, which leads to the promotion of apoptosis, including elevated BAX expression. (4) In OVCAR‐3 cells specifically, PCG induces autophagy. The “Overall Outcome” panel illustrates that these combined mechanisms lead to significant anti‐proliferative and anti‐metastatic effects, highlighting PCG's potential as a therapeutic strategy for ovarian cancer.

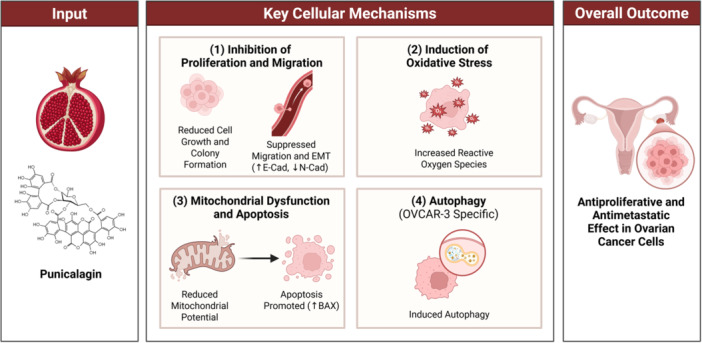

## 1 Introduction

Ovarian cancer (OC) is the second most common gynecological cancer and is a significant cause of morbidity and mortality among women, especially in developed countries [1, 2]. It is the leading cause of gynecological cancer‐related deaths globally, with an estimated 20,890 new cases and 12,730 deaths reported in 2025 in the USA [2]. OC is usually asymptomatic in the early stages, making detection difficult. There are three main types of ovarian cancer, namely epithelial ovarian cell cancer (EOC), stromal cell cancer, and germ cell cancer, of which EOC is the most common and accounts for 90% of all OCs. Germ‐cell ovarian cancer usually occurs in the young age group, while sex‐cord stromal ovarian cancer develops in older individuals. The occurrence of OC is higher in postmenopausal women and women of advanced age [3, 4]. The progression of ovarian cancer can be divided into four stages. In stage I, the cancer is localized to the ovaries alone. In stage II, the cancer spreads to the pelvic area. In Stage III, there is involvement of the lymph nodes or peritoneal regions, followed by Stage IV, which represents the most serious condition with distant organ metastasis [3].

Currently, the standard interventions to halt ovarian cancer growth involve surgical resection, followed by a chemotherapeutic regimen of platinum‐based agents and paclitaxel [5]. Initially, 80 percent of patients exhibit a favorable response to treatment; yet, paradoxically, recurrence and the development of medication resistance are commonly observed. Patients on these chemotherapeutic regimens frequently experience cumulative toxic effects, adversely impacting their quality of life, and the 5‐year survival rate remains notably low [6]. This is due to the capacity of cancer stem cells (CSCs) to develop resistance to chemotherapy and reinitiate tumor proliferation and spread [7]. Consequently, there is a need to investigate alternative pharmaceutical agents or innovative therapeutic approaches for the treatment of ovarian cancer.

Substances that are not traditionally regarded as standard therapy are a part of complementary and alternative medicine (CAM) [8, 9]. CAM encompasses a wide range of practices aimed at reducing cancer risk, including the utilization of phytochemicals found in food, produced both naturally and artificially. CAM covers a broad spectrum of medical practices, from homeopathic treatment to the use of dietary supplements and essential oils. Epidemiological and preclinical research suggests that some cancer types may be substantially influenced by dietary and behavioral variables. As a result, phytochemicals in food have increased in popularity. Numerous phytochemicals in the diet exhibit bioactive properties that may be useful for cancer prevention [8].

Pomegranate, or *Punica granatum*, is a member of the *Punicaceae* family. Researchers have discovered that the leaves of the pomegranate tree have antioxidant, anti‐inflammatory, and anti‐cancer properties, which contribute to their extensive use in traditional medicine. Pomegranates have two main types of polyphenolic compounds: anthocyanins and hydrolyzable tannins. These polyphenols account for 90% of the antioxidant activity in pomegranates; punicalagin (PCG), a hydrolyzable tannin found in pomegranate leaves and bark, alone accounts for about 50% of this activity [10]. Reportedly, PCG is also reported to have anti‐inflammatory and anticancer activity, among others [11, 12]. PCG has been shown in previous research to have therapeutic effects on various types of cancer, including leukemia and osteosarcoma [11]. The efficacy of PCG is yet to be ascertained in ovarian cancer cells.

Apoptosis is a form of cell death that is controlled by the intrinsic and extrinsic pathways [13, 14]. Apoptosis is initiated early on in embryonic development as a means of eliminating defective cells. The extrinsic pathway is triggered as a reaction to signals from outside the cell, while the intrinsic pathway is responsible for cell death caused by internal stress. The two routes work together to activate caspases, which cause cell death by the indiscriminate breakdown of proteins. Apoptosis is an important mechanism to prevent cancer cell proliferation. Blocking apoptosis causes cells to divide uncontrollably, which can develop into cancer.

Epithelial cells undergo a genetic transformation known as epithelial‐mesenchymal transition (EMT), during which they become capable of migration and invasion while losing their polarity and tight connections, including cell‐cell adhesion and cell‐extracellular matrix adhesion [15]. This enhances their motility and gives them the ability to invade. Cell invasion and migration of neoplastic epithelial cells occur due to EMT [16].

This study investigated the efficacy of PCG in two biologically distinct epithelial ovarian cancer cell lines, SKOV‐3 and OVCAR‐3, selected to capture differences in invasiveness, molecular background, and therapeutic responsiveness. PCG was found to be cytotoxic to ovarian cancer cells. PCG also inhibited ovarian cancer cell invasion in vitro. Moreover, the PCG inhibited the metastasis of ovarian cancer cells by modulating the E‐cadherin and N‐cadherin proteins.

## 2 Materials and Methods

### 2.1 Reagents and Chemicals

A stock solution of the 80 mM PCG was maintained in the lab by dissolving it in dimethyl sulfoxide (DMSO). PCG was purchased from ChemCruz (Santa Cruz Biotechnology Inc., Dallas, USA).

### 2.2 Cell Culture and Media

Ovarian cancer cell lines SKOV‐3 and OVCAR‐3 were obtained from ATCC (Manassas, USA). DMEM supplemented with FBS (10%), antibiotics (1%), and HEPES (1%) was used to maintain the cells growth throughout the experiments. Furthermore, these cells were provided with a humid environment containing 5% CO_2_ at the 37°C during the trial period. On reaching the confluence of 70%–80% (after every 3 days), the cells were sub‐cultured using the 1% Trypsin‐EDTA (Invitrogen Life Technologies Inc., USA) for 3 min. SKOV‐3 and OVCAR‐3 cells were selected to represent distinct biological and molecular characteristics of epithelial ovarian cancer. SKOV‐3 cells are relatively more invasive, display chemoresistant features, and harbor p53 dysfunction, whereas OVCAR‐3 cells are less migratory and exhibit different genetic and phenotypic profiles. The use of these two cell lines allowed us to evaluate the consistency of PCG effects across biologically distinct ovarian cancer models.

### 2.3 Cell Viability Assay

Ovarian cancer cells were subjected to WST‐8 assay [17] to check the viability of cells at various doses at different time intervals after treating the cells with PCG. Briefly, a total of 3 × 10^3^ cells were seeded in a 96‐well plate for 24 h. After that, the cells were treated with different doses of PCG (0, 6.25, 12.5, 25, 50, 100, and 200 µM) for a total time duration of 24, 48, and 72 h. Later on, the cells viability was determined by Quanti‐MAX WST‐8 Cell Viability Assay Kit (Biomax, Guri‐si, Korea) as per the manufacturer's guidelines and the absorbance at 450 nm by employing the Synergy NEO 2 multimode plate reader (BioTek, Winooski, USA).

### 2.4 Colony Formation Assay

The cells were seeded in the 6 well plates at a density of 200 cells per well and incubated for 24 h prior to treating the cells with PCG. After treatment, the cells were further incubated for 48 h. After 48 h, the fresh media was maintained in the cell culture wells for 14 days. Later, the cells were fixed using 100% methanol (for 20 min) and stained with a 0.5% crystal violet solution (for 15 min) [18]. Lastly, the wells were washed using DPBS.

### 2.5 Wound Healing Assay

The wound healing assay is a commonly used in vitro method to assess how drugs influence cell migration [19]. OVCAR‐3 and SKOV‐3 cells were seeded in 6 well plates for 24 h. After achieving the confluence of 80%–90%, the cell surface was scratched with a 1‐mL pipette tip. The wells were washed, filled with 1% FBS, and incubated. The wounds created were analyzed after 24 and 48 h with a phase‐contrast microscope. The rate of cell migration was calculated by analyzing the scratched wound area. The area was then measured using Fiji (NIH, Bethesda, USA). All of the experiments were conducted in triplicate to ensure the accuracy of the data.

### 2.6 Transwell Migration Assay

The transwell chamber (24‐well, 8.0 µm pore membrane) was used for this study [20]. Approximately 4 × 10^3^ cells per well were seeded in the upper chamber in 250 μL of serum‐free medium, and 750 µL of serum‐free medium was added to the lower chamber. After 48 h, the cells in the upper chamber were removed using cotton swabs and fixed and stained using 4% paraformaldehyde and a 0.1% crystal violet solution. The cells penetrated through the membrane and were detected using the fluorescence microscope.

### 2.7 JC‐10 Assay

Ovarian cancer cells were seeded in the 96‐well black plate at a density of 4 × 10^3^ cells per well for 24 h prior to treatment with PCG. After treating the cells with PCG, the plates were incubated again for 48 h. Subsequently, the cells were treated with 5 µM JC‐10 (AdipoGen Life Sciences, San Diego, USA) in DMSO and incubated for 30 min [21]. After that, the plates were quickly transferred to the Lionheart FX Automated Microscope (BioTek, Winooski, USA) to detect the distribution in mitochondrial membrane potential.

### 2.8 MitoSOX Assay

The MitoSOX assay was also used in this study to detect the formation of ROS species in the SKOV‐3 and OVCAR‐3 cells [22]. Briefly, the cells were seeded at 4 × 10^3^ cells per well in a 96 well black plate for 24 h, and then for 48 h after treating the cells with different doses of PCG. Later on, we treated the cells with MitoSOX (5 μM) and Hoechst33342 (10 μg/mL) for 20 min and detected the formation of ROS using the Lionheart FX Automated Microscope (BioTek, Winooski, USA).

### 2.9 Acridine Orange Staining

To detect the presence of acidic vesicular organelles (AVOs), indicative of autophagy, acridine orange staining was used [18]. Briefly, after the initial incubation of 24 h, the cells were treated with various doses of PCG for 48 h. Cells were provided with 5% CO_2_ and 37°C during the incubation. After that, the cells were treated with acridine orange staining (1 µg/mL) for 20 min. Lastly, the images were captured using the Lionheart FX Automated Microscope (BioTek, Winooski, USA) after 2 washes with DPBS. Images captured were further analyzed using the Gen5 v 3.14.03 software.

### 2.10 Flow Cytometry Analysis

For the analysis of apoptosis [23], ovarian cancer cells were seeded in the 6‐ well plate and incubated for 24 h. Later on, the cells were treated with different doses of PCG for 48 h. After that, the rate of Apoptosis was detected using the Annexin V‐FITC Apoptosis Detection Kit (Sigma‐Aldrich, St. Louis, USA). Briefly, the cells collected were centrifuged and treated as per the manufacturer's guidelines. Cells were incubated in the dark for 15 min at 4°C before the detection of stained cells by using BD FACSymphony A3 Cell Analyzer (BD Bioscience, San Jose, USA). Data obtained was analyzed using the FlowJo v. 10.5.3 software.

### 2.11 Western Blot Analysis

For the western blot analysis [24], the SKOV‐3 and OVCAR‐3 cells were treated with 100 and 200 μM for 24 h. After this, the cells were scraped using a cell scraper and centrifuged for 5 min at 300 rpm The cells were then mixed with PRO‐PREP Protein Extraction Solution (C/T) (iNtRON Biotechnology, Seongnam‐si, Korea) and kept at −20°C for 20 min. Later on, the cells were centrifuged at 13,000 rpm for 5 min, and the supernatant containing the extracted protein was used for SDS page gel run after quantification of protein using the BCA protein assay. Primary antibodies, listed in Table 1, were used to detect the proteins after the transfer of proteins to a PVDF membrane. The membrane was blocked using the 5% skim milk before treating the PVDF membrane with primary and secondary antibodies. GAPDH was used as a control protein in this study. Lastly, Cytiva Amesham ECL start Western blotting detection reagent was used to detect the protein in the Lumino Graph 2 (ATTO, Tokyo, Japan). Fiji (Fiji is just ImageJ 1.54f) was used for the quantification of the bands obtained.

**Table 1:** List of antibodies used in Western blot assay.

Protein	Manufacturer	Identifier	Source/Description	Dilution
BAX	Cell Signaling Technology	5023	Rabbit mAb	1:1000
E‐cadherin	Cell Signaling Technology	3195	Rabbit mAb	1:1000
N‐cadherin	Cell Signaling Technology	13116	Rabbit mAb	1:1000
LC3A/B	Cell Signaling Technology	12741	Rabbit mAb	1:1000
GAPDH	Cell Signaling Technology	4970	Rabbit mAb	1:1000

### 2.12 Statistical Analysis

All experiments were performed in at least three independent biological replicates, defined as experiments conducted on separate days using independently cultured cells. Data were analyzed using GraphPad Prism 10.4.1 (GraphPad Software Inc., San Diego, USA). Statistical comparisons were performed using one‐way ANOVA followed by Dunnett's multiple comparison test. Data are presented as mean ± SD unless otherwise specified in the figure legends. Exact *p*‐values are provided in the figure legends where feasible, and *p* < 0.05 was considered statistically significant.

## 3 Results

### 3.1 Anti‐Proliferative Effects of PCG on Ovarian Cancer Cells

**Figure 1:**
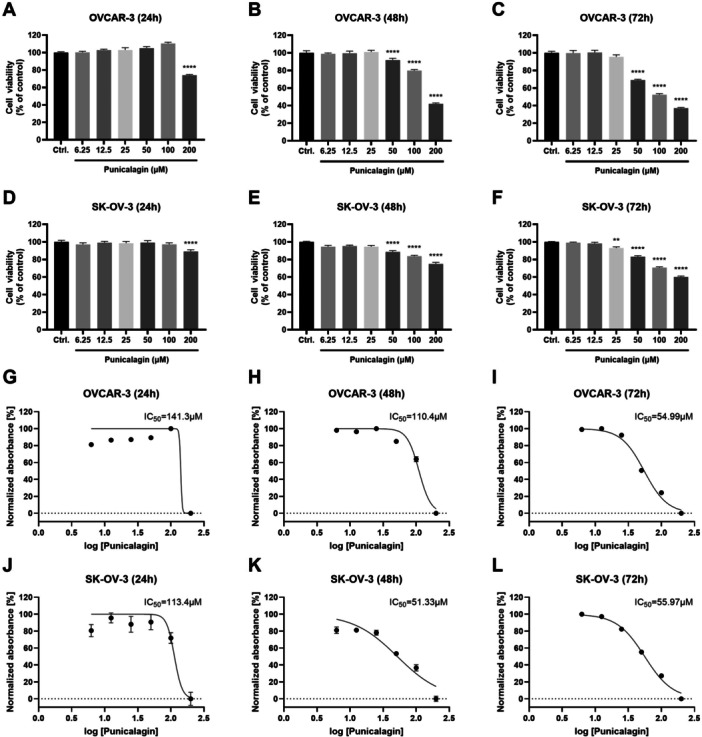
Punicalagin dose‐dependently decreases the growth of ovarian cancer cells. The ovarian cancer cell lines, OVCAR‐3 and SKOV‐3, were treated with PCG (from 6.25 to 200 µM) for 24, 48, and 72 h. Cell proliferation was detected by the WST assay. PCG caused a decrease in the growth of (A–C) OVCAR 3 cells and (D–F) SKOV‐3 cells with varying IC_50_s. IC_50_ dose concentrations are presented in (G–I) for OVCAR‐3 cells and (J–L) SKOV‐3 cells. Following the statistical analysis, the data in the graphs have been derived from at least three repeated experiments and shown as the mean ± SD. *p ≤ 0.05 compared to control.

The ovarian cancer cells, OVCAR‐3 and SKOV‐3, were treated with various doses of PCG (0, 6.25, 12.5, 25, 50, 100, and 200 µM) for 24, 48, and 72 h. The viability of cancer cells was evaluated using the water‐soluble tetrazolium salt (WST) assay. PCG reduced the growth of ovarian cancer cells in a dose and time‐dependent manner (Figure 1A–F). The half‐maximal inhibitory concentrations (IC_50_s) at different time intervals were statistically different for the two cell lines. The IC_50_ values for the OVCAR‐3 cells at 24, 48, and 72 h were found to be 141.3, 110.4, and 54.99 µM, respectively (Figure 1G–I). Similarly, the IC_50_ values for the SKOV‐3 cells were 113.4, 51.33, and 55.97 µM for 24, 48, and 72 h, respectively (Figure 1J–L). With the increase in time, the effectiveness of PCG at lower doses increased and vice versa. These results indicate the antiproliferative activity of PCG against ovarian cancer cells.

### 3.2 Inhibitory Effects of PCG on the Colony Forming Ability of Ovarian Cancer Cells

**Figure 2:**
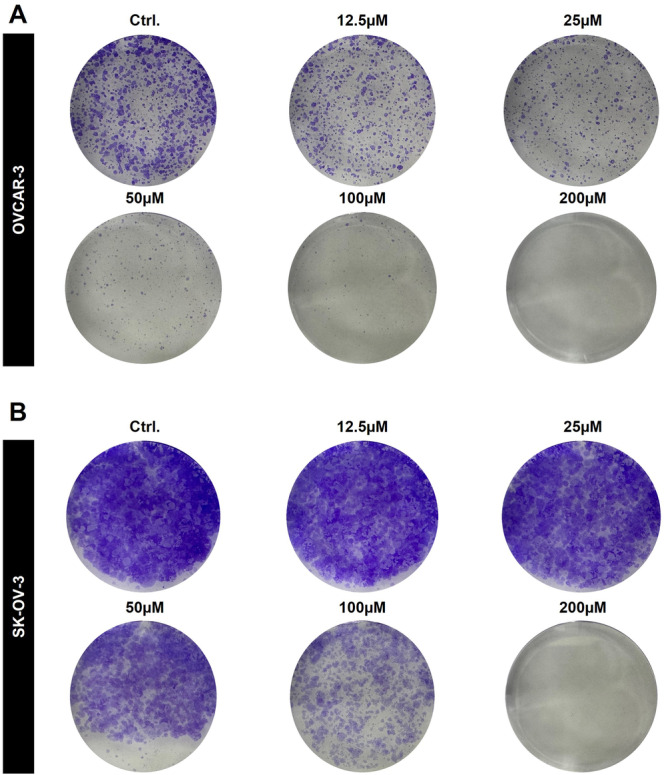
Punicalagin suppressed the colony formation ability of ovarian cancer cells. The ovarian cancer cell lines, OVCAR‐3 and SKOV‐3, were treated with PCG (from 6.25 to 200 µM) for 48 h and then incubated for 14 days. PCG causes a decrease in the colony formation ability of the (A) OVCAR‐3 and (B) SKOV‐3 cells. The inhibitory effect was more prominent in the OVCAR‐3 as compared to SKOV‐3 cells.

The OVAR‐3 and SKOV‐3 cells were treated with PCG doses ranging from 12.5 to 200 µM for 48 h, and then the cells were cultured for another 14 days. There was a dose‐dependent decrease in the number of colonies of both cell lines. The efficacy of PCG on OVCAR‐3 cells (Figure 2A) was more prominent at lower doses when compared with the SKOV‐3 cells (Figure 2B). Higher doses of PCG (50, 100, and 200 µM) showed a more significant effect in SKOV‐3 cells when compared to lower doses of PCG. This experiment indicated the inhibitory effect of PCG on the colony‐forming ability of ovarian cancer cells.

### 3.3 Inhibitory Effect of PCG on the Migratory Ability of Ovarian Cancer Cells

**Figure 3:**
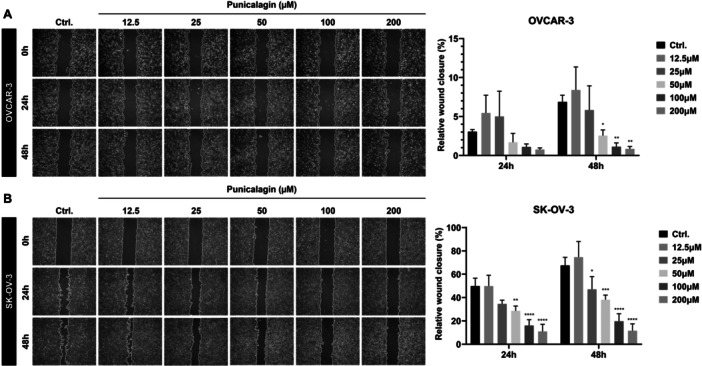
Punicalagin decreased the migration ability of ovarian cancer cells. The wound healing assay showed a decrease in the ability of ovarian cancer cells to migrate after PCG treatment. PCG caused such an effect in 48 h in (A) OVCAR‐3 cells and for 24 and 48 h in (B) SKOV‐3 cells. The highest cell migration inhibitory effects were obtained in the SKOV‐3 cells as compared to OVCAR‐3 cells. Data were obtained after statistical analysis using at least three repeated experiments, presented in graphs as the mean ± SD. *p ≤ 0.05 compared to control.

**Figure 4:**
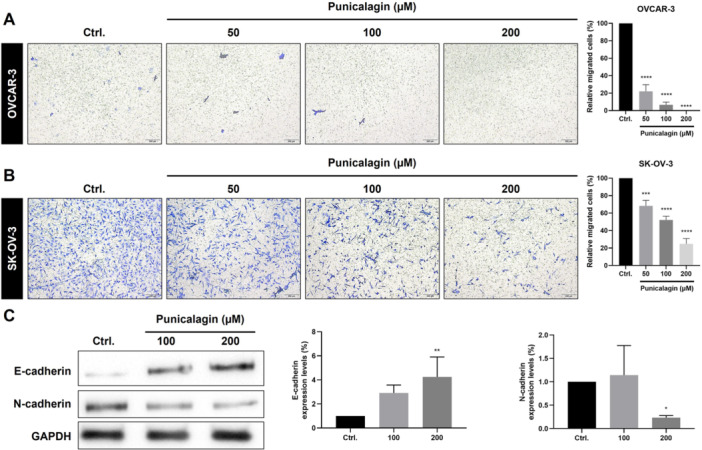
Punicalagin suppressed the transwell migration ability of ovarian cancer cells. To assess the transwell migration ability of PCG on the ovarian cancer cells, the OVCAR‐3 and SKOV‐3 were subjected to 48 h to PCG at various doses (50, 100, and 200 µM). PCG treatment resulted in the decreased transwell migration ability of the (A) OCAR‐3 and (B) SKOV‐3 cells. (C) Western blot assay revealed a decrease in the N‐cadherin, while an increase in E‐cadherin was observed in SKOV‐3 cancer cells. Data were obtained after statistical analysis using at least three repeated experiments, presented in graphs as the mean ± SD. *p ≤ 0.05 compared to control.

To investigate whether PCG reduces the migration ability of ovarian cancer cells, a wound‐healing assay was performed in OVCAR‐3 and SKOV‐3 cells. It was found that the PCG inhibited the migration ability of OVCAR‐3 cells after 48 h of treatment at 50, 100, and 200 µM (Figure 3A). However, PCG was found to be more effective against SKOV‐3 cells, wherein PCG reduced the migration of cells at both 24 h (50, 100, and 200 µM) and 48 h (25, 50, 100, and 200 µM) (Figure 3B). Furthermore, a transwell migration assay was also used to assess the ability of cells to migrate through the transwell membrane after PCG treatment. The results of the transwell migration assay were similar to the wound healing assay (Figure 4A,B). PCG (50, 100, and 200 µM) was able to successfully inhibit the transwell migration ability of both OVCAR‐3 and SKOV‐3 cell lines. The SKOV‐3 cell line is more invasive compared to the OVCAR‐3 cell line. The effects of PCG in inhibiting wound migration and transwell migration were significantly better in the SKOV‐3 cell line compared to OVCAR‐3 [25]. Furthermore, the results were further analyzed using the Western blot assay. It was found that PCG increased the expression of E‐cadherin and decreased the expression of N‐cadherin in SKOV‐3 cells (Figure 4C). In summary, PCG was found to inhibit the metastatic ability of ovarian cancer cells. Notably, the concentrations of PCG used in the migration assays overlap with those that significantly reduced cell viability and increased apoptosis. Therefore, we cannot exclude the possibility that the observed reduction in wound closure and transwell migration is partially attributable to decreased proliferation and/or increased cytotoxicity rather than a direct anti‐migratory effect.

### 3.4 PCG Disrupts Mitochondrial Function and Increases Reactive Oxygen Species Levels in Ovarian Cancer Cells

**Figure 5:**
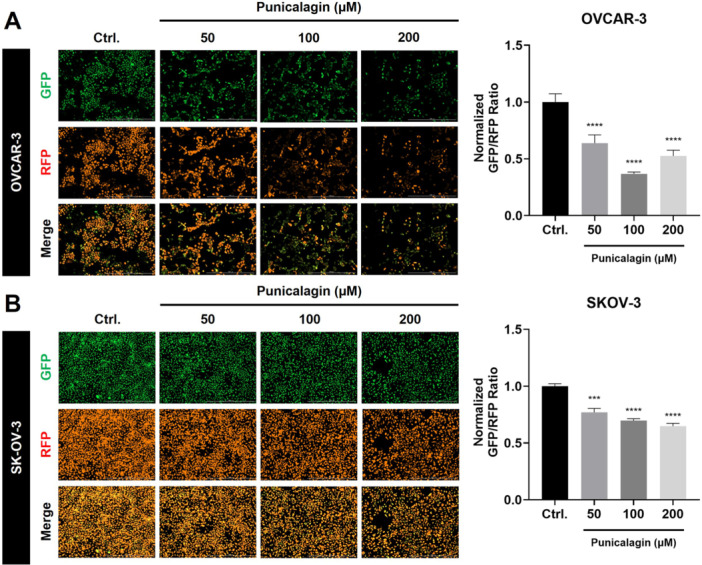
Punicalagin disrupted the mitochondrial membrane potential in ovarian cancer cells. Representative images for the analysis of MMP through the JC‐10 assay in (A) OVCAR‐3 cells and (B) SKOV‐3 cells. All cells were seeded at a density of 4 × 10^3^ per well in cell culture dishes and treated with (0.1% DMSO) and PCG (50, 100, and 200 µM). Data were obtained after statistical analysis using at least three repeated experiments, presented in graphs as the mean ± SD. *p ≤ 0.05 compared to control.

**Figure 6:**
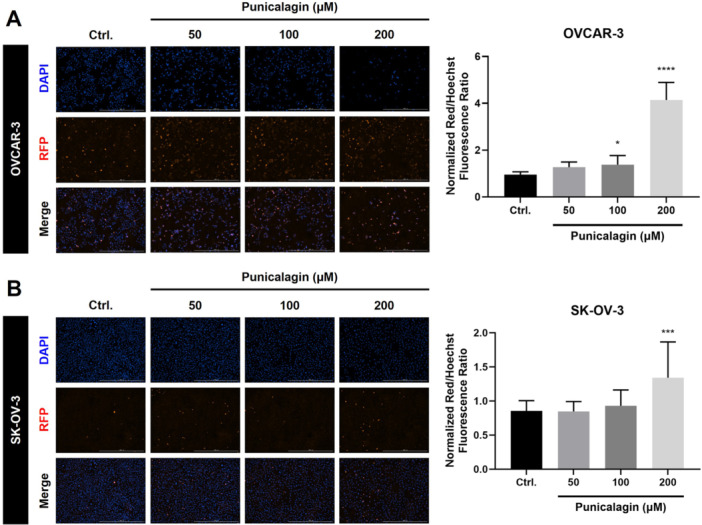
Punicalagin caused an increase in ROS production in ovarian cancer cells. For the quantification of the ROS, the ovarian cancer cells were subjected to PCG for 48 h before employing the MitoSOX assay. There was an increase in the ROS generation at higher doses for (A) OVCAR‐3 and (B) SKOV‐3 cells. Data were obtained after statistical analysis using at least three repeated experiments, presented in graphs as the mean ± SD. *p ≤ 0.05 compared to control.

It is well known that the mitochondrial membrane potential (MMP) plays a vital role in the cell death mechanism of cancer cells [26]. A JC‐10 assay was performed to detect the changes in the MMP after PCG treatment given to the OVCAR‐3 and SKOV‐3 cell lines for 48 h. The PCG treatment decreased the MMP of the cancer cells, which was consistent for both OVCAR‐3 (Figure 5A) and SKOV‐3 cells (Figure 5B). Furthermore, reactive oxygen species (ROS) play an important role in the anticancer activity of various anticancer drugs. Increased ROS has been reported to trigger apoptosis, necroptosis, ferroptosis, autophagy, and other cell death processes in cancer cells [27]. As expected, ROS levels in both ovarian cancer cell lines increased after treatment with higher doses (50, 100, and 200 µM) of PCG (Figure 6A,B). These findings demonstrate that PCG treatment leads to increased ROS production and a decrease in the MMP. Unfortunately, this does not demonstrate a direct mechanistic role of ROS in mediating the PCG‐mediated cytotoxicity, as antioxidant rescue experiments were not performed.

### 3.5 Proapoptotic Effects of PCG on Ovarian Cancer Cells

**Figure 7:**
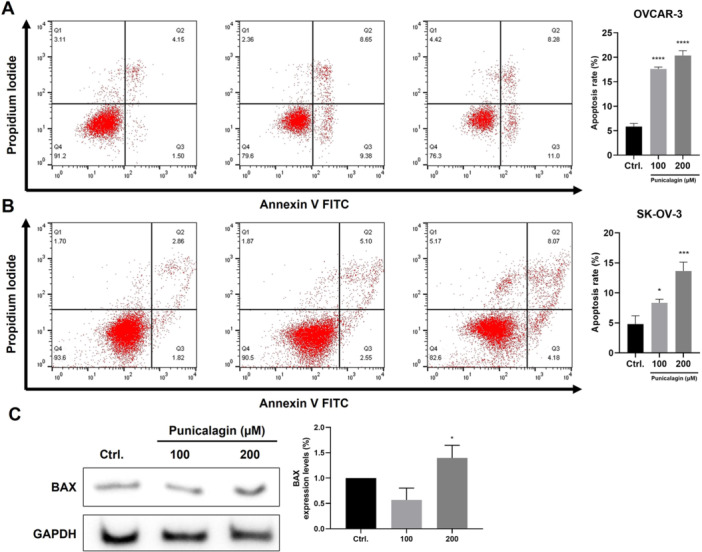
Punicalagin caused apoptosis in ovarian cancer cells. Representative images for the analysis of Annexin V/PI assay in (A) OVCAR‐3 and (B) SKOV‐3 cells. All cells were seeded at a density of 8 × 10^4^ per well in cell culture dishes and treated with (0.1% DMSO) and PCG (50, 100, and 200 µM). (C) Representative image of the BAX expression in the SKOV‐3 cells. Data were obtained after statistical analysis using at least three repeated experiments, presented in graphs as the mean ± SD. *p ≤ 0.05 compared to control.

After confirmation of the antiproliferative effect of PCG on ovarian cancer cells, the mechanism of cell death was investigated. Apoptosis was quantified using flow cytometry after staining the cells using Annexin V/Propidium iodide (PI). It was found that treatment with PCG caused dose‐dependent apoptosis of the OVCAR‐3 and SKOV‐3 cells (Figure 7A). The apoptosis rate was more dose‐dependent in the SKOV‐3 cells as compared to the OVCAR‐3 cells. Moreover, a western blot analysis revealed increased levels of the proapoptotic BCL2‐associated X, apoptosis regulator (BAX) protein (Figure 7B). These results suggest that PCG treatment may promote apoptosis and BAX expression in ovarian cancer cells. The executioner events downstream of BAX via caspase activation or PARP cleavage were not studied in this experiment.

### 3.6 Effect of Punicalagin on Autophagy in Ovarian Cancer Cells

**Figure 8:**
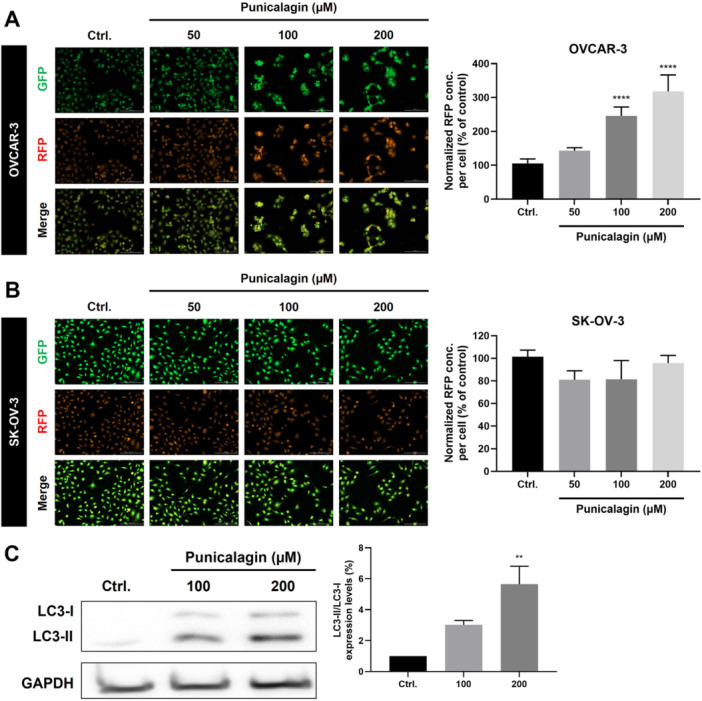
Punicalagin causes autophagy in ovarian cancer cells. Representative images for the analysis of autophagy through Acridine Orange staining in (A) OVCAR‐3 and (B) SKOV‐3 cells. All cells were seeded at a density of 4 × 10^3^ per well in cell culture dishes and treated with (0.1% DMSO) and PCG (50, 100, and 200 µM). Punicalagin caused autophagy in the OVCAR‐3 cell line. (C) Representative image of the BAX expression in the OVCAR‐3 cells. Data were obtained after statistical analysis using at least three repeated experiments, presented in graphs as the mean ± SD. *p ≤ 0.05 compared to control.

Acridine orange (AO) staining was utilized to identify if PCG treatment resulted in autophagy. The formation of red vesicles after AO staining was detected by fluorescence microscopy. Treatment with PCG resulted in autophagy in the OVCAR‐3 cells (Figure 8A). However, no such effect was seen with SKOV‐3 cells (Figure 8B). Furthermore, the western blot assay also revealed a conversion of LC3I to LC3II in OVCAR‐3 cells after 24 h of treatment, indicating that PCG is able to trigger autophagy to inhibit the proliferation of OVCAR‐3 cells (Figure 8C). These findings indicate that PCG treatment is associated with the accumulation of acidic vesicular organelles and increased LC3‐II levels in OVCAR‐3 cells, suggesting induction of autophagy‐related processes. However, as autophagic flux assays (e.g., lysosomal inhibition or p62/SQSTM1 turnover) were not performed, these data reflect static autophagy markers and do not establish functional autophagic flux or a direct role of autophagy in cell death.

## 4 Discussion

Natural compounds have been tested for their anticancer activities in various cancers, including ovarian cancer. PCG, the active ingredient of pomegranate, has been identified as having anti‐tumor [10], antimicrobial [28], and anti‐inflammatory [29] properties. The anti‐cancer efficacy of PCG has been tested earlier in different cancers, including thyroid, lung, breast, colorectal, and cervical cancers [11]. However, the mechanism involved in inhibiting the growth of ovarian cancer cells is not known. Hence, the current study was planned to address this lacuna. In this study, we found that PCG inhibits the growth of ovarian cancer (SKOV‐3 and OVCAR‐3) cells. Our results showed that PCG treatment induced apoptosis in both OVCAR‐3 and SKOV‐3 cells and was associated with autophagy‐related changes in OVCAR‐3 cells, as evidenced by LC3‐I to LC3‐II conversion and acridine orange staining.

ROS are known to regulate multiple oncogenic signaling pathways, including WNT/β‐catenin, Keap1/Nrf2/ARE, Notch, and PI3K/AKT/mTOR. However, these pathways were not directly examined in the present study. The excessive accumulation of ROS leads to cellular oxidative stress (OS), causing disturbances in cell division. Moreover, ROS accumulation in the tumor microenvironment (TME) establishes an immunosuppressive milieu that in turn facilitates the initiation and progression of ovarian cancer via its impact on the immune cells. However, when ROS levels surpass a critical threshold, they exert cytotoxic effects, leading to oxidative DNA damage, apoptosis, and inhibition of tumor growth. This concentration‐dependent duality of ROS has been exploited in cancer therapy. For example, curcumin, a natural flavonoid obtained from the *Curcuma longa*, was found to inhibit the growth of colorectal cancer cells via ROS generation by the KEAP1/NRF2/microRNA (miR)−34a/b/c pathways [30]. Furthermore, polyphyllin II, a common saponin obtained from *Paris polyphylla*, suppressed the growth of glioma cells by ROS generation. This then led to the BAX/cytochrome c (Cyt‐c) apoptosis in these cells [31]. Similarly, PCG has also been found to increase ROS generation in various cancer cells, such as gastric [32], bladder [33], breast [10], and lung [34] cancer cells. These earlier findings support a potential link between oxidative stress and reduced tumor cell proliferation. In our study, PCG treatment increased ROS levels concomitant with reduced growth and proliferation of both SKOV‐3 and OVCAR‐3 cells. However, since antioxidant inhibition or rescue experiments were not performed, our data demonstrate an association rather than a direct mechanistic requirement for ROS in mediating these effects. The same was true for the MMP, which was decreased when cells were treated with PCG at the tested concentrations when compared to the control group. In healthy cells, JC‐10 forms “J‐aggregates” displaying red fluorescence. In contrast, as the membrane potential decreases, JC‐10 monomers are produced, resulting in a shift to green fluorescence [35]. These findings suggest that PCG treatment is associated with increased ROS levels and decreased mitochondrial membrane potential. While previous reports indicate that ROS can influence many downstream signaling pathways, we have not directly examined specific signaling cascades in this study.

Patients with metastatic cancer have a drastically lower 5‐year survival rate compared to those with localized tumors [36]. Cancer metastasis to other organs causes death in about 90% of cancer patients [37]. Metastasis is a complex and continuously changing process that starts with the uncontrolled transformation of normal cells into oncogenic cells. Once transformed, these oncogenic cells acquire traits that allow them to resist programmed cell death, initiate angiogenesis, invade local tissues, remain in circulation, and eventually spread to different organs [38]. Previous studies have reported that PCG modulates NF‐κB signaling in other cancer types [39]. However, NF‐κB or related signaling pathways were not assessed in the current study. In our experiments, PCG reduced migration‐associated behaviors and modulated EMT‐related markers (E‐cadherin and N‐cadherin) in ovarian cancer cells. Furthermore, because these assays were performed at concentrations that also reduced cell viability and increased apoptosis, the observed decrease in migration may be partially secondary to cytotoxic and anti‐proliferative effects rather than solely reflecting a direct inhibition of migratory capacity. Moreover, the differential responses observed between SKOV‐3 and OVCAR‐3 cells further underscore the importance of employing multiple ovarian cancer models. SKOV‐3 cells are generally considered more invasive and display distinct molecular alterations compared to OVCAR‐3 cells, which may partially explain differences in migration inhibition and autophagy‐related responses. The inclusion of both cell lines strengthens the robustness and generalizability of our findings.

Apoptosis, or programmed cell death, takes place in cells for different reasons. It is a way to suppress tumor development by triggering apoptosis. In this case, mutant cells are usually recognized by immune‐system cells, which trigger death signaling in these cells. The reaction may determine the competing ability of pre‐cancerous and malignant cells to respond to the apoptotic signal, depending largely on the expression of the pro‐apoptosis and survival genes [40]. In many recent studies, PCG was able to cause apoptosis in cancerous cells. PCG caused apoptosis by an increase in the regulation of BAX and caspase‐3 expression in breast cancer cells [41]. Likewise, PCG caused apoptosis in human gastric cancer cells by activating caspase‐3 by suppressing the phosphorylation of extracellular signal‐regulated kinase (Erk), inhibitor of nuclear factor‐κB (IκB) kinase (IKK), and NF‐κB [32]. Consistent with previous studies, we observed an increase in Annexin V–positive cells and elevated BAX expression following PCG treatment. These findings support the induction of apoptosis; however, we did not evaluate downstream executioner events such as caspase‐3 activation or PARP cleavage. Therefore, the precise apoptotic signaling cascade activated by PCG in ovarian cancer cells remains to be further defined.

Autophagy, a self‐defense system, protects cells from various stresses, oxidative pressure, and hypoxia by recycling damaged organelles and proteins to generate constituent amino acids [42]. The regulation of autophagy is pivotal to ensuring the optimal anticancer mechanisms of therapeutic agents inducing oxidative stress. Recent studies have indicated that PCG may activate autophagy as a proapoptotic or preventive signal in order to limit the proliferative capacity of malignant cells [43, 44]. One feature of autophagy conserved throughout evolution is the formation of the double‐membrane autophagosome [45]. Autophagy is induced in cells by extrinsic stimuli. These chemical agents include chemotherapeutic reagents and ionizing radiations that could induce cancer cells to live or die, though this depends on the type of cancer being treated, the nature of the stimulus, the concentration of that stimulus, and the duration of treatment [46]. Furthermore, autophagy is considered a protective process. However, under certain extreme conditions, autophagy could lead to cell death [47]. It was observed that PCG treatment increased the LC3‐II/LC3‐I ratio and acridine orange–positive vesicular structures in OVCAR‐3 cells, suggesting activation of autophagy‐related pathways. However, autophagic flux and the functional contribution of autophagy to cell viability were not examined using pharmacologic or genetic inhibition approaches. Therefore, we cannot conclude whether autophagy plays a cytoprotective or cytotoxic role in this context. However, no such effect was observed in the SKOV‐3 cells, which can be attributed to a defect in the autophagy regulators, such as p53, which play an important role in both autophagy and apoptosis [48].

An additional limitation of this study is the relatively high concentrations of PCG (50–200 µM) used in several mechanistic assays. While these concentrations were selected based on IC_50_ values and are consistent with prior in vitro studies investigating punicalagin, they may exceed physiologically achievable plasma levels following dietary intake or conventional administration. Therefore, the translational relevance of these findings should be interpreted with caution. Future studies evaluating lower, pharmacokinetically achievable concentrations, as well as in vivo models incorporating bioavailability and metabolism considerations, will be necessary to determine the clinical applicability of PCG.

In the present study, vehicle‐treated cells (0.1% DMSO) served as the negative control across all experiments. A dedicated positive control agent (e.g., cisplatin, paclitaxel, or staurosporine) was not included, as our primary objective was to systematically characterize the dose‐ and time‐dependent effects of PCG across multiple functional assays under standardized conditions. While inclusion of a positive control could have provided benchmarking relative to established cytotoxic agents, prior studies have demonstrated that PCG reduces viability, induces apoptosis, and suppresses migration‐related behaviors in ovarian cancer models, including A2780 cells, using comparable in vitro readouts [49]. These published findings support the biological activity of PCG in ovarian cancer; nevertheless, direct comparative studies with standard‐of‐care agents in SKOV‐3 and OVCAR‐3 models will be important in future investigations to strengthen translational interpretation.

Another limitation of the present study is the absence of a normal ovarian epithelial or non‐transformed control cell line. As a result, we were unable to evaluate the selectivity of PCG toward malignant versus non‐malignant cells. Assessment of therapeutic selectivity is critical for translational development, and future studies should include normal ovarian epithelial cells or other appropriate non‐tumorigenic controls to determine whether the observed cytotoxic effects are cancer‐specific.

In conclusion, PCG suppresses proliferation and migration‐associated behaviors and increases apoptotic markers in biologically distinct ovarian cancer cell lines, accompanied by ROS accumulation and mitochondrial membrane potential disruption. Autophagy‐related changes were observed in OVCAR‐3 cells; however, their functional contribution to cytotoxicity remains to be defined. Collectively, these findings support the potential of punicalagin as a bioactive compound targeting multiple stress‐response pathways in ovarian cancer. Future studies should focus on defining the mechanistic requirement of ROS and autophagy, evaluating pharmacologically achievable concentrations, assessing selectivity in non‐malignant ovarian epithelial models, and validating anti‐tumor efficacy in vivo to determine translational feasibility.

## Author Contributions

**Zeeshan Ahmad Bhutta:** methodology, validation, formal analysis, investigation, data curation, writing – original draft, visualization. **Cho‐Won Kim:** methodology, validation, formal analysis, investigation, writing – review and editing, visualization. **Hwayoung Na:** methodology, validation, formal analysis, data curation, visualization. **Hong Kyu Lee:** methodology, validation, formal analysis, investigation, writing – review and editing. **Kyung‐Chul Choi:** conceptualization, data curation, writing – review and editing, supervision, project administration, funding acquisition.

## Conflicts of Interest

The authors declare no conflicts of interest.

## Data Availability

The data that support the findings of this study are available from the corresponding author upon reasonable request.
